# Digital Health in Melanoma Posttreatment Care in Rural and Remote Australia: Systematic Review

**DOI:** 10.2196/11547

**Published:** 2018-09-24

**Authors:** Audrey Rollin, Brad Ridout, Andrew Campbell

**Affiliations:** 1 Faculty of Health Sciences The University of Sydney Sydney Australia

**Keywords:** digital health, eHealth, technology, melanoma, posttreatment care, support care services, rural areas, remote communities, patient-centric, oncology

## Abstract

**Background:**

The melanoma incidence and mortality rates in rural and remote communities are exponentially higher than in urban areas. Digital health could be used to close the urban/rural gap for melanoma and improve access to posttreatment and support care services.

**Objective:**

The aim of this review was to understand how digital health is currently used for melanoma posttreatment care and determine the benefits for Australian rural and remote areas.

**Methods:**

A systematic search of PubMed, Medline, PsycINFO, and Scopus was conducted in March 2018. Findings were clustered per type of intervention and related direct outcomes.

**Results:**

Five studies met the inclusion criteria, but none investigated the benefits of digital health for melanoma posttreatment care in rural and remote areas of Australia. Some empirical studies demonstrated consumers’ acceptance of digital intervention for posttreatment care. The findings did not take into consideration individual, psychological, and socioeconomic factors, even though studies show their significant impacts on melanoma quality of aftercare.

**Conclusions:**

Digital interventions may be used as an adjunct service by clinicians during melanoma posttreatment care, especially in regions that are less-resourced by practitioners and health infrastructure, such as rural and remote Australia. Technology could be used to reduce the disparity in melanoma incidence, mortality rates, and accessibility to posttreatment care management between urban and rural/remote populations.

## Introduction

Australia remains a country with one of the highest levels of melanoma. In 2015, the worldwide average age-standardized incidence rate (ASR) for melanoma was 5 cases for 100,000. However, the rates for Australia and New Zealand are over ten times that level (Table 1) [1]. The high incidence of melanoma in Australia and New Zealand—whose populations consist primarily of transplanted, fair skinned, northern Europeans—is due to high levels of ambient ultraviolet (UV) radiation. Exposure of the skin to UV radiation is a well-known risk factor for melanoma [2-3]. Melanoma treatment represents a significant cost for the Australian Health Care System that has increased dramatically in the past two decades, from approximately Aus $30 million in 2001 to Aus $201 million in 2017 [4].

**Table 1:** Worldwide ranking of the average age-standardized incidence rate for melanoma.

Rank	Country	Age-standardized incidence rate for melanoma (95% CI)
1	New Zealand	54/100,000 (39-73)
2	Australia	54/100,000 (41-78)
3	Norway	26/100,000 (18-32)
4	Sweden	26/100,000 (20-35)
5	The Netherlands	25/100,000 (17-30)

Cutaneous melanoma (CM) is the fourth most commonly diagnosed cancer in Australia [5] and the most common cancer among young Australians between 15-39 years old. Although melanoma represents only 2% of all skin cancers [6], it often leads to premature death [6] and is responsible for a majority of skin cancer deaths [7]. Compared to urban populations, Australia’s rural and remote communities experience inequities in access to care [8], leading to a higher incidence and mortality within 5 years. The median incidence ASR for nonindigenous Australians with CM is 32 per 100,000 across rural and remote areas and 27 per 100,000 in major cities. In comparison, the median worldwide ARS mortality for CM is 5.4 per 100,000 across rural and remote areas and 4.6 per 100,000 in major cities [9].

Melanoma treatment plans depend on (1) prognostic factors which are primarily defined by the American Joint Committee on Cancer staging system [10], and (2) individual characteristics which will allow the clinicians to determine the type of melanoma and the risk for recurrences. For example, patients previously treated for primary CM are at higher risk of recurrences and developing new primary melanomas and skin lesions [11]. However, early detection can reduce mortality rates, as melanoma can be more effectively cured with simple and inexpensive treatments in the early stages [12]. In 1996, Berwick and colleagues [13] reported that total skin self-examination (TSSE) might decrease melanoma mortality by 63%. In 2003, the study by Carli et al [14] found that regular skin self-examination (SSE) could significantly reduce the likelihood of a tumor >1 mm thick at diagnosis. It has been suggested that early detection is a factor influencing the disparity between urban and rural survival rates, but other aspects such as access to health services, clinical practices, and medical care management need to be taken into consideration to fully evaluate survival rates, especially after an initial diagnosis and treatment for CM [15].

In 2017, the Australian Institute of Health and Welfare estimated that 14,000 new melanoma cases would be diagnosed. However, there are only 775 registered dermatologists in Australia (only 260 of which are melanoma specialists), and very few of them are easily accessible to people living in rural and remote areas [16]. There are several infrastructure, cost, and access limitations which impact on the provision of health services for people. This is further compounded by the lack of training for future dermatologists and general practitioners (GPs) in remote areas.

It has been suggested that technology-based training and telehealth could help combat this disparity by bringing health services to rural and remote areas [17]. Many studies have evaluated the benefits of eHealth and the level of acceptance for digital intervention in the early detection of cutaneous melanoma [18-20]. Benefits of telemedicine and teledermatology include increased access to health care services, reduced travel and waiting times, and cost-effectiveness [19]. A 2006 study by Qureshi et al [21] reported that patients prefer telemedicine if it can provide quicker access to their physicians. However, a qualitative review found that patients’ attitudes toward technology are only positive if the tool is personalized and adapted to the recipients’ needs and characteristics [18]. Also, available evidence suggests that telemedicine is not only beneficial for patients, but for health care professionals (HCP) too. For example, a previous study by Al-Qirim [22] reported that GPs appreciate using teledermatology when they need to refer to a dermatologists’ expertise in order to obtain a second opinion.

In order to structure posttreatment plans, physicians must refer to the clinician guidelines. A recent study [23] showed that clinicians working with rural populations are less likely to properly apply guidelines when it comes to educating patients towards surveillance and supportive care. For example, patients living in rural areas were less likely to be provided with patient education material (86% compared to 89% in urban areas) or encouraged to conduct SSE (86% compared to 81%). There are also concerns that oral educational information provided by clinicians may not be useful. A study by Damude et al [24] found that only 5% of melanoma patients were able to reproduce all 4 critical characteristics of their tumor correctly. These results suggest a need for better quality and greater consistency in providing information to patients.

An area of posttreatment care that is often neglected across all populations is psychosocial support. Psychological distress, including worry, anxiety, and fear of disease recurrences and death, are common for survivors [25,26]. However, only 1% of specialists suggested patients see a psychologist as part of their post-treatment plan, despite an entire chapter of the clinician guidelines being devoted to psychosocial issues related to melanoma [23].

Although reviews have evaluated the effectiveness of technology for melanoma early detection, no studies have directly highlighted the benefits of eHealth on melanoma posttreatment care for rural communities. Researchers have qualitatively examined the different forms of treatment and care between rural and urban populations [27] and the care needs among rural cancer patients [28]. However, these studies did not focus on melanoma posttreatment care.

It is unclear from the published literature the level and utility of technology support available to patients with melanoma living in remote areas. The primary aim of this systematic review was to (1) examine how technology is currently used and accepted by physicians and patients with melanoma, and (2) to determine if there has been any implementation of such systems in rural and remote areas of Australia. With this focus, the researchers seek to identify areas of weakness and bring to light hypotheses on how technology could be used as an adjunct service during posttreatment care of CM, to aid physicians in designing follow-up care plans for patients with CM based on their needs and personal characteristics.

## Methods

### Databases and Search Strategy

The overall aim of this systematic review was to investigate digital health acceptance and its current use among people treated for melanoma. Our primary aim was to better understand digital health benefits among rural and remote populations for CM. However, given the impact of CM across all of Australia’s population, literature around digital health and CM that impacted urban and regional areas was incorporated as well. This was done to ensure broad inclusion of digital health practice for CM posttreatment care. The databases selected were searched using keyword combinations related to digital health and melanoma posttreatment care. Specifically, we used the keyword combination “telehealth” OR “telemedicine” OR “teledermatology” OR “online services” OR “ehealth” OR “e-health” OR “eHealth” AND “melanoma.” For the current systematic literature review, 4 databases (PubMed, Medline, PsycINFO, Scopus) were searched in March 2018.

### Study Selection

The search was limited to peer-reviewed papers. Search results identified 451 papers which were exported into a Microsoft Excel document. After duplicates were removed, 271 articles remained.

**Figure 1:**
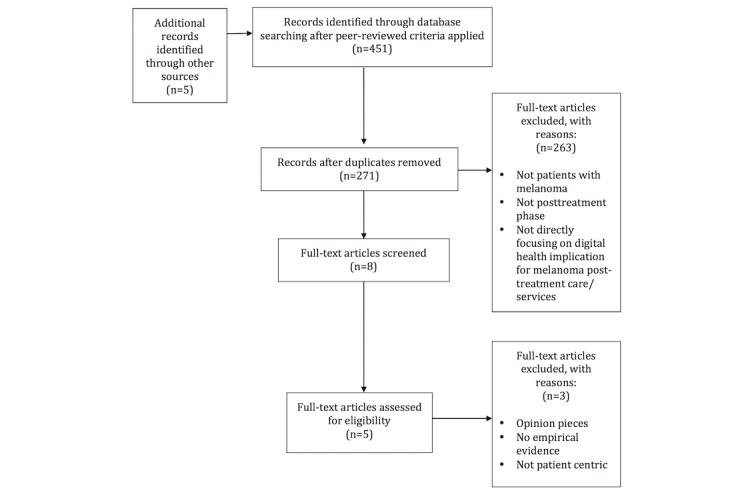
Preferred Reporting Items for Systematic Reviews and Meta-Analyses (PRISMA) flowchart of the systematic literature review.

The search strategy involved 2 screening phases. Each article was screened based on exclusion criteria to remove irrelevant articles from the initial selection of 271 articles. For the second phase, only studies that were based on empirical evidence and used a patient-centric approach were retained for the final systematic literature review. Figure 1 presents the selection overview based on the Preferred Reporting Items for Systematic Reviews and Meta-Analyses (PRISMA) flowchart. A PRISMA checklist is shown in Multimedia Appendix 1.

### Data Extraction

Data was extracted from the relevant papers using the following classification: (1) sources (country, year of study intervention), (2) participant characteristics (gender, residential area, mean ages, patient illness conditions, level of education, and socioeconomic background), (3) study design, (4) study intervention, and (5) research focus (Multimedia Appendix 1).

## Results

### Origin

There were 5 studies in total. Two (40%) of the studies were from Scotland, with the other 3 (60%) from the Netherlands, Canada, and the US. All studies were from before 2015 except for one (20%) study from the Netherlands, which was from 2016.

### Participant Characteristics

Four of the 5 (80%) studies consisted of patients with melanoma only. The remaining study (20%) recruited patients with a history of melanoma and psoriasis, or collateral cancer. A minority, 2 of the 5 (40%) authors referred to the patient’s illness condition in their sample description. The gender distribution of studies was mostly homogeneous with 47%-60% males and a mean age ranging from 53-66 years. None of the studies used “residential area” as an independent variable. Two (40%) studies used residential area as a patient characteristic but did not mention it in their findings. Also, 2 (40%) studies reported socioeconomic criteria in their findings and 3 (60%) featured level of education.

### Study Design and Intervention

**Figure 2:**
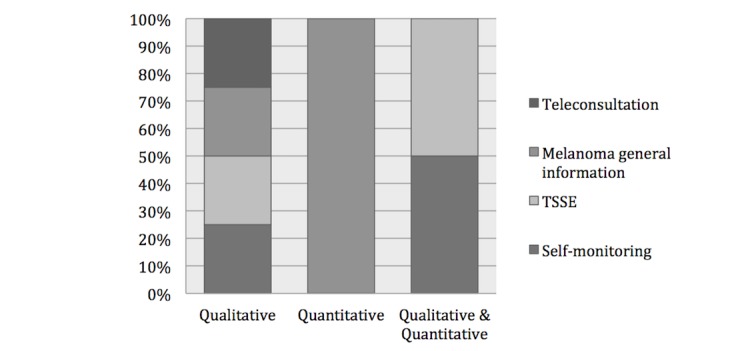
Distribution of the papers according to the study design and the main focus area. TSSE: total skin self-examination.

Prior to the investigation, all published research participants were informed of the objectives of the studies. Three of the 5 (60%) studies [18,19,21] were qualitative and used semistructured interviews either face-to-face or over the phone. The interviews were recorded by the researchers, transcribed verbatim, coded and reviewed by 1 or more coresearchers in order to cluster by themes/concepts of the participants’ answers. The 3 (60%) qualitative studies assessed the perception and preferences of dermatology patients about the use of technology for self-monitoring and TSSE [18], a Web-based platform (Oncology Interactive Navigator) to deliver information about melanoma [19], and store and forward teleconsultation [21]. The latter used a willingness-to-pay approach in order to investigate dermatology patients’ preferences. One (20%) study [20] used both qualitative and quantitative methods to assess the feasibility and acceptability of a digital intervention for self-monitoring and the participants’ attitude to perform TSSE. One quantitative study [24] used an online questionnaire in order to capture participants’ knowledge of melanoma and TSSE, and their preferences. Figure 2 displays the study design distribution with regards to the research main focus areas.

### Research Focus Areas

Table 2 presents the positive and negative outcomes of using technology for melanoma posttreatment care of each selected study by type of intervention. The studies reviewed were classified under four intervention categories: (1) total skin self-examination; (2) teleconsultation; (3) clinicians’ support and coordination; and (4) informative and supportive displays.

**Table 2:** Direct outcomes on posttreatment care per type of intervention.

Direct outcomes				Type of intervention	
**Total skin self-examination**					
	**Positive findings**				
		Provides reassurance to patients [18]			Report sent by phone to clinicians including photographs Self-monitoring supportive tools
		Convenient Avoids in-person clinical visit if not necessary [18]			Report sent by phone to clinicians including photographs
		Reduces the number of people who might forget about total skin self-examination [18]			Reminder sent by text message or email
		Promotes early detection [18]			Report sent by phone to clinicians including photographs
		Behavior change Empowers patients’ confidence to perform total skin self-examination [20,21]			Self-monitoring supportive tools YouTube videos explaining how to perform a total skin self-examination
		Reinforces total skin self-examination [20]			Self-monitoring supportive tools
	**Negative findings**				
		Health care professionals based their opinion on pictures only [18]	Clinicians’ feedback sent by text message or email		
**Teleconsultation**					
	**Positive findings**				
		Convenient Reduces travel and saves time [18,20] Quick access to clinicians [18,21]	Skype or teleconference Store and forward telemedicine		
	**Negative findings**				
		Patients’ desire to discuss face-to-face with clinicians [18]	Skype or teleconference		
		Patients’ skin required to be examined by clinicians [18]	Phone		
**Clinicians’ support and coordination**					
	**Positive findings**				
		Accuracy in the diagnosis [18]	Three-way consultation via a video or Skype link from the general practitioner’s room		
		Convenient Time and travel saved [15]	Remote point of contact Nurse specialist’ opinion to be provided via store and forward system		
	**Negative findings**				
	Not applicable		Not applicable		
**Informative and supportive displays**					
	**Positive findings**				
		Promotes early detection [18,19]	Web-based app tailored information delivered about their conditions Skin map		
		Reduces patients’ stress [19]	Web-based app tailored information delivered about their conditions Skin map		
		Improves patients’ decision-making in treatment [19]	Web-based app tailored information delivered about their conditions		
		Ease of communication Content is more adapted to the patients’ level of understanding [19] Supporting oral/written information delivered to the patients [24]	Web-based app tailored information delivered about their conditions YouTube videos explaining how to perform a total skin self-examination		
		Reduce/control the content load [18,19]	Web-based app tailored information delivered about their conditions		
	**Negative findings**				
		Don't want to be associated with other patients Makes them feel sicker than they are [18]	Online peer support (ie, forum, group chat)		
		Do not replace the oral and written information provided by clinicians [24]	YouTube videos explaining how to perform a total skin self-examination		

## Discussion

### Principal Results

The primary aim of this review was to identify the different use of digital health for melanoma posttreatment care, including its benefits and weaknesses. Patients perceived digital health as an added value to their posttreatment care [18-21,24]. However, a majority of the studies reported the benefits of digital interventions to prevent recurrence and promote early detection [18,19,24]. None of the selected studies investigated the benefits of digital health for melanoma posttreatment care in rural and remote areas. This gap in the digital health literature gives thought to a very specific niche in telemedicine that needs to be explored further, given this is an at-risk population [5]. Thus, it is crucial to understand how digital health could help clinicians to provide better care and quality of life (QoL) for people treated with melanoma, especially in regions where aftercare resources are limited or nonexistent, such as in rural and remote areas of Australia.

### Patients’ Individual Characteristics

This review found some evidence for the efficacy of digital interventions for melanoma posttreatment care. Key findings identified that clinicians need to take into consideration patients’ characteristics in order to provide personalized follow-up plans, tailored information, and quality of care [18,21]. It is clear that information technology (IT) capabilities, patient age, illness condition, level of incomes and residential areas influence clinician and patient decision-making in the posttreatment plan. One study by Hall and Murchie [18] found that participants who were familiar with technology and not living close to hospitals were more likely to have a positive attitude toward telemedicine for self-monitoring and performing TSSE [18]. Querish and colleagues [21] also reported that 73% of the participants are more willing to pay when telemedicine was giving them faster access to the clinicians. Among this sample, 55% had an income inferior, or equal to US $50,000 per annum. Another study [29] investigating consumers’ perception toward telemedicine found that people with “technology anxiety” were less likely to use IT for specific care. In contrast, young populations may be more inclined to trust digital health interventions, as they are more familiar with technology [30].

### Patients’ Acceptance

In order to efficiently use personal consumer technology in melanoma posttreatment care, it is crucial to understand patients’ acceptance toward digital intervention. Several of the studies reviewed [18,20,24] illustrated a shift from “passive” recipients to “active” patients for their care [19], which led to proactive health behavior change and positive attitudes toward early detection. Simple measures such as receiving a reminder to perform TSSE by text message or email, having access to informative videos, or using smartphone apps for self-monitoring, reduced anxiety, and reinforced TSSE [18,20,24]. These technologies could also be used to address the need for better quality and greater consistency in information provided to melanoma patients [24].

The study by Quereshi and colleagues [21] reported that patients’ attitude toward telemedicine was generally positive if it showed convenience (58% well willing to pay up to US $125), but almost universally positive if it gave a quicker access to their clinicians (95% of the patients were willing to pay up to US $500). The study by Horsham and colleagues [30] emphasized that survivors show a positive attitude towards a digital health application that allowed them to monitor QoL and provided tailored information and advice.

While these findings demonstrated that patients were generally receptive toward digital health for melanoma posttreatment care, no studies to date have focused on rural and remote communities’ views. Nevertheless, a few studies have already highlighted people’s acceptance toward telemedicine in Australian rural and remote communities for cancer more broadly. In their studies, Sebesan and colleagues [31,32] reported the benefits of teleoncology in rural and remote areas for cancer care. The main benefits of this telehealth system included travel time saved and better access to specialist care. Also, studies [32,33] have shown that telehealth may lead to financial benefits and improved quality of care in distant communities.

### Patients’ Psychological and Social Needs

In this systematic review, there was a lack of empirical evidence with regards to the benefits of digital health for support and psychological care services, in order to provide better QoL. These studies mainly focused on early detection, including self-monitoring and TSSE. However, a previous systematic review [34] suggested that 30% of patients with melanoma reported psychological distress, which interferes with QoL, medical cost, risk of recurrence, and mortality rates [35,36]. Likewise, Oliveria and colleagues [37] found that patients treated with melanoma showed (1) direct psychosocial concerns related to conducting skin self-examination, (2) anxiety associated with new recurrence and sun exposure, (3) familial concerns, and (4) financial constraints and maintenance of health insurance benefits. Emotional support and reassurance are considered a key component of care [34-40], with psychological intervention associated with superior survival and recurrence rates, and decreased distress [39]. Clinicians should, therefore, take into consideration the psychosocial impact on patient outcomes when designing posttreatment plans.

### The Economic Burden of Melanoma Treatment in Australia

Melanoma early detection reduces the mortality rate and results in simple treatments for lower cost [41]. A 2017 study [2], estimated the mean cost to the Australian health system for melanoma treatment to be Aus $10,716 per patient. However, treatment cost for advanced melanoma may be 21% to 70% more expensive than for early stages (in situ, stage I and stage II). Doran and colleagues [42] compared the direct and indirect costs of melanoma and nonmelanoma skin cancer (NMSC) in 2010. The direct costs related to the management of the disease, including diagnosis and treatment to follow-up, and indirect costs included productivity losses associated with morbidity and premature mortality. Estimates of direct lifetime cost per case were Aus $10,230 for melanoma and Aus $2336 for NMSC; and total indirect cost per case Aus $34,567 for melanoma and Aus $123 for NMSC.

Moreover, additional studies [15,27] have reported an urban and rural disparity in term of accessing health care and mortality rate. Yu and colleagues [27] reported that socioeconomic factors may impact people’s decision-making in selecting their health care provider. The study showed a difference in provider performance based on patients’ income. Rural populations with lower-income received poorer care from HCPs, compared to patients living in urban areas.

The comparatively lower cost of delivering support care services via digital health initiatives, in addition to reduced treatment costs associated with promoting early detection [17-19] would go some way to improving access to health care and reduce urban/rural inequity.

### Limitations

This systematic literature review presents several limitations. First, most of the studies used small samples (n≥20). It is evident that digital health research regarding melanoma postcare treatment is still in its early stages of investigation. Second, few studies were identified as focusing on the psychosocial and health economic side of post-care treatment, as melanoma studies are primarily focused on early detection, and those that did use a retrospective measurement of consumer attitudes towards telemedicine. Third, melanoma treatment plans depend on individual characteristics, including the disease staging. Only one of the studies used staging as a participant characteristic. Finally, although the authors were primarily interested in rural and remote areas of Australia, the lack of studies conducted in these areas meant that studies for this review were drawn from across the world, and their conclusions may not necessarily generalize to the Australian rural and remote context.

Overall, the current systematic review provides findings of patients’ perceptions toward telemedicine and digital interventions already used by clinicians and patients. However, in order to have a complete review of digital health benefits for melanoma post-treatment care, it would have been necessary to look at HCP’s acceptance of such technological interventions.

### Conclusion

The study of digital health has become an area of focus in primary health care, as it can help clinicians in their practice and support patients in improving and monitoring their QoL. While there is research interest in using digital health in early detection of melanoma, there is an urgent need to explore the potential for benefits of digital health in melanoma post-treatment care for specific needs and intervention, particularly for rural and remote populations who are lagging behind regarding postcare treatment quality and availability. This literature review also highlights the importance of considering individual, psychosocial and socioeconomic characteristics in future developments in this area.

Although our findings showed positive outcomes with regards to using technology during post-treatment care, there were also some limitations in using digital health. Patients believe that technology cannot replace the clinician provided written and oral information, follow-up visits, or clinical interventions [24]. To summarize, digital health shows potential to be used as an adjunct service by clinicians during melanoma posttreatment care, especially in regions that are less-resourced by practitioners and health infrastructure, such as regional and remote Australia.

### Implication for Further Research

Future research should explore the potential for digital health within rural and remote areas for melanoma posttreatment care in order to reduce the mortality rate disparity in between urban and rural populations. Also, it will be interesting to consider how digital health implementation may transform the patients’ ecosystem and the cost-effectiveness of this solution for both patients and the health care industry.

Interdisciplinary studies in behavioral psychology and health economy can add new insights to the health care industry in term of benefits and services that digital health can bring to melanoma patients care in rural and remote areas.

## Appendix

Multimedia Appendix 1 Preferred Reporting Items for Systematic Reviews and Meta-Analyses (PRISMA) Checklist.

Multimedia Appendix 2 The consumer-technology relationship and digital interventions for melanoma posttreatment care.

## Abbreviations

ASR: age-standardized rate
CM: cutaneous melanoma
GPs: general practitioners
HCP: health care professional
IT: information technology
NMSC: nonmelanoma skin cancer
QoL: quality of life
SSE: skin self-examination
TSSE: total skin self-examination
UV: ultraviolet
